# Aggressive Retinopathy of Prematurity in a Larger and Less Preterm Infant: A Review of Possible Risk Factors

**DOI:** 10.7759/cureus.19267

**Published:** 2021-11-04

**Authors:** Siti Noor Atikah Abd Rahman, Safinaz Mohd Khialdin, Shareena Ishak

**Affiliations:** 1 Ophthalmology, Hospital Universiti Kebangsaan Malaysia, Kuala Lumpur, MYS; 2 Pediatrics, Hospital Universiti Kebangsaan Malaysia, Kuala Lumpur, MYS

**Keywords:** pediatric ophthalmology, low birth weight, retinopathy of prematurity, aggressive retinopathy, preterm infant

## Abstract

Aggressive retinopathy of prematurity (A-ROP), formerly known as aggressive posterior retinopathy of prematurity (APROP), occurs generally in extremely premature infants less than 28 weeks gestational age with extreme low birth weight of ≤1000g. We report a case of A-ROP occurring in a larger and less preterm infant. The possible risk factors that lead to the occurrence of A-ROP in this infant will be discussed.

An infant born vaginally at 30 weeks gestational age weighing 1550g was diagnosed with A-ROP. Retinopathy of prematurity (ROP) screening was performed due to the presence of risk factors: prematurity, low birth weight, received supplemental oxygen, intraventricular hemorrhage and history of maternal chorioamnionitis. Following a single injection of intravitreal ranibizumab, significant regression of A-ROP was observed. A-ROP was unexpected in this infant and was believed to have developed as a result of receipt of supplemental oxygen, maternal chorioamnionitis, and *Ureaplasma *infection.

## Introduction

Retinopathy of prematurity (ROP) is a vision-threatening disease and a well-known cause of childhood blindness worldwide. It is estimated that blindness caused by this disease occurred in at least 50,000 children globally [1]. In a survey of 24 blind schools in Malaysia in 2012, 17.4% had childhood blindness or severe visual impairment due to ROP [2]. Higher survival rate of more premature babies as a result of improved neonatal care may have led to an increased incidence of ROP. Prior to the very recent consensus by the International Classification of Retinopathy of Prematurity Third Edition, a rapidly progressive and more severe stage of ROP was aggressive posterior retinopathy of prematurity (APROP), which typically developed in extreme premature infants [3]. However, since this severe form of ROP is increasingly occurring in larger preterm infants, a new term of aggressive retinopathy of prematurity (A-ROP) is recommended. A-ROP is characterized by pathologic neovascularization that develops rapidly associated with severe plus disease and the progression does not follow the classic stages of ROP [4]. On the other hand, APROP is featured by its location at posterior retina in zone 1 or posterior zone 2 [3], otherwise similar pathologic vascularization and plus disease. A-ROP is different from APROP in terms of the location of the disease that may extend beyond the posterior retina. Because of the similar appearance of vascular abnormalities with APROP, we will discuss the possible risk factors with references to APROP. Known risk factors for APROP include extreme prematurity less than 28 weeks gestational age (GA) [3], extreme low birth weight (ELBW) less than 1000g [5], and prolonged unmonitored oxygen therapy [6]. Other risk factors reported include thrombocytopenia, multiple infections, and necrotizing enterocolitis [7]. Maternal risk factor such as chorioamnionitis has been reported to be associated with the development of APROP [8]. We report a case of A-ROP, which was previously known as APROP, occurring in a larger and less premature infant receiving treatment in our hospital. We will discuss the possible risk factors which lead to the development of A-ROP in this patient.

## Case presentation

A premature male infant at 30 weeks period of gestation was delivered via vaginal delivery with birth weight of 1550 grams. His Apgar score was 6 and 9 at one and five minutes, respectively. He had respiratory distress syndrome at birth and required invasive ventilator support for 14 hours. He received a dose of intra-tracheal surfactant therapy. He was subsequently extubated to non-invasive ventilator support for the next 18 days. His highest oxygen requirement while receiving ventilator assistance was 30%. On day 19 of life, he was placed on low oxygen therapy at 1L/min which was subsequently weaned off at day 27 of life. At seven weeks of life, he was diagnosed with *Ureaplasma urealyticum* pneumonia and completed 14 days of oral azithromycin therapy.

Serial cranial ultrasounds performed during his stay in the Neonatal Intensive Care Unit showed presence of bilateral intraventricular hemorrhage grade II, which subsequently resolved by two months of age. He was also diagnosed with gastro-esophageal reflux disease (GERD) which was confirmed with a pH study. As a result, he had recurrent episodes of significant apnoea.

His mother was diagnosed with chorioamnionitis prior to delivery. She had preterm premature rupture of membrane (PPROM) associated with fever. High vaginal swab culture grew Group B Streptococcus (GBS). However culture of placenta was negative and histopathological examination of the placenta did not reveal significant abnormality.

**Figure 1:**
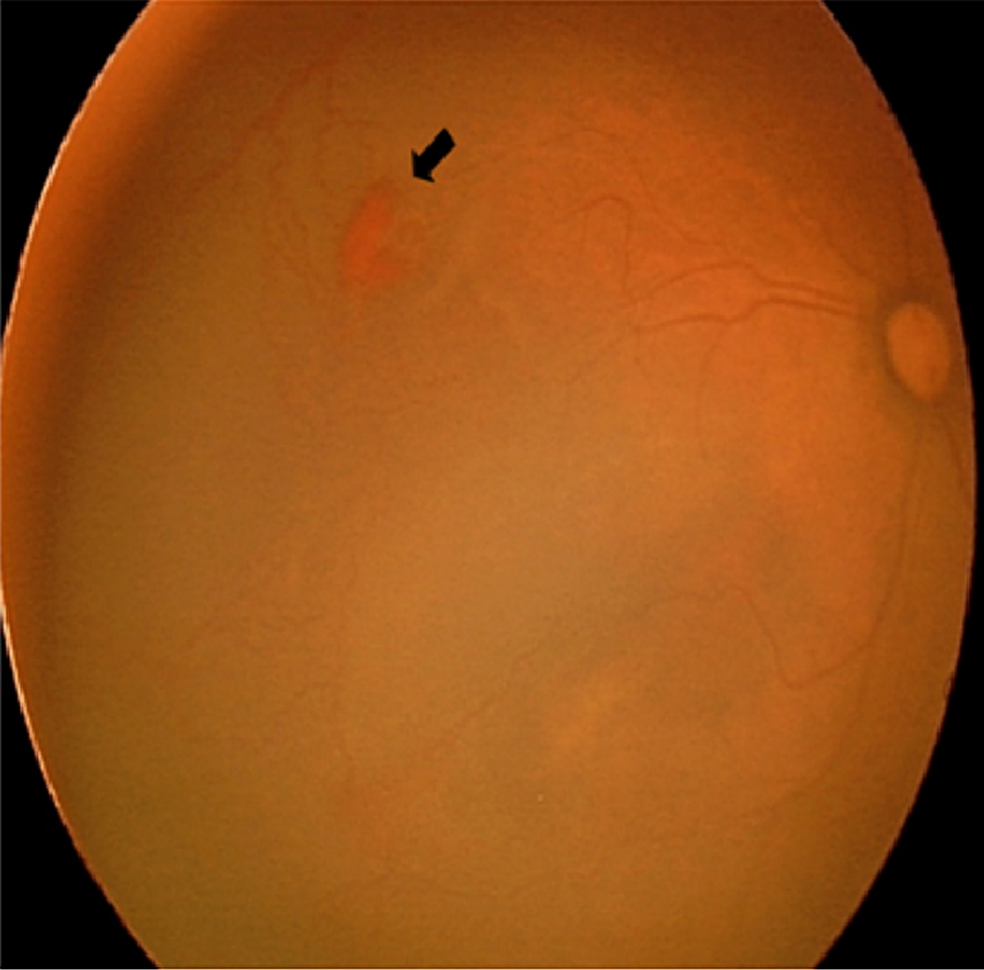
Fundus of the right eye showed pre-retinal hemorrhage (black arrow) about 1 disc diameter size at posterior zone 2, with dilated and tortuous vessels seen at temporal 10 o’clock

**Figure 2:**
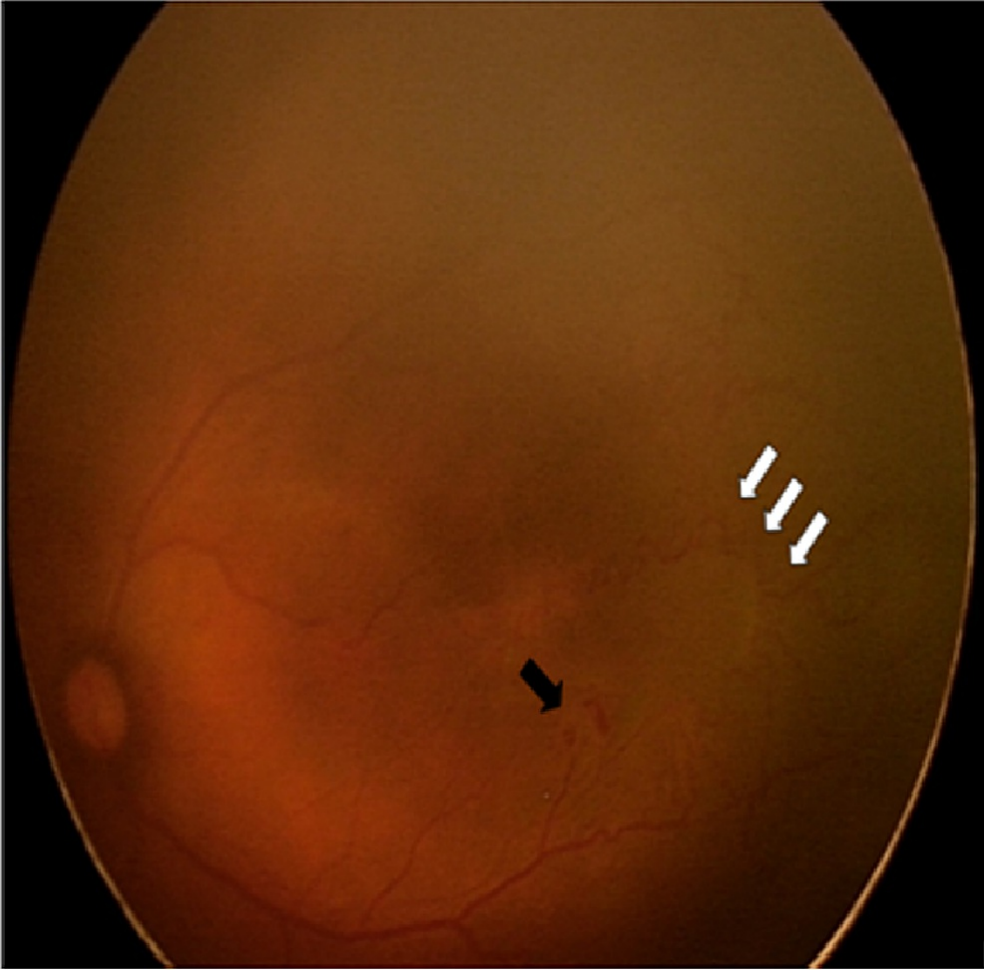
Fundus of the left eye showed pre-retinal hemorrhage (black arrow) at 4 o’clock at posterior zone 2. Arteriovenous shunting resembling vascular loop can be seen (white arrow) with dilated and tortuous vessels

The baby was screened for ROP due to the presence of risk factors. Upon review at 34 weeks and five days corrected gestational age (CGA), anterior segments of both eyes were normal and pupils were well dilated pharmacologically. However, fundus examination revealed pre-retinal hemorrhage at posterior zone 2, nonphysiologic vascular loop, arteriovenous shunt and dilated and tortuous vessels in both eyes (Figures 1, 2). There was no demarcation line or ridge seen.

**Figure 3:**
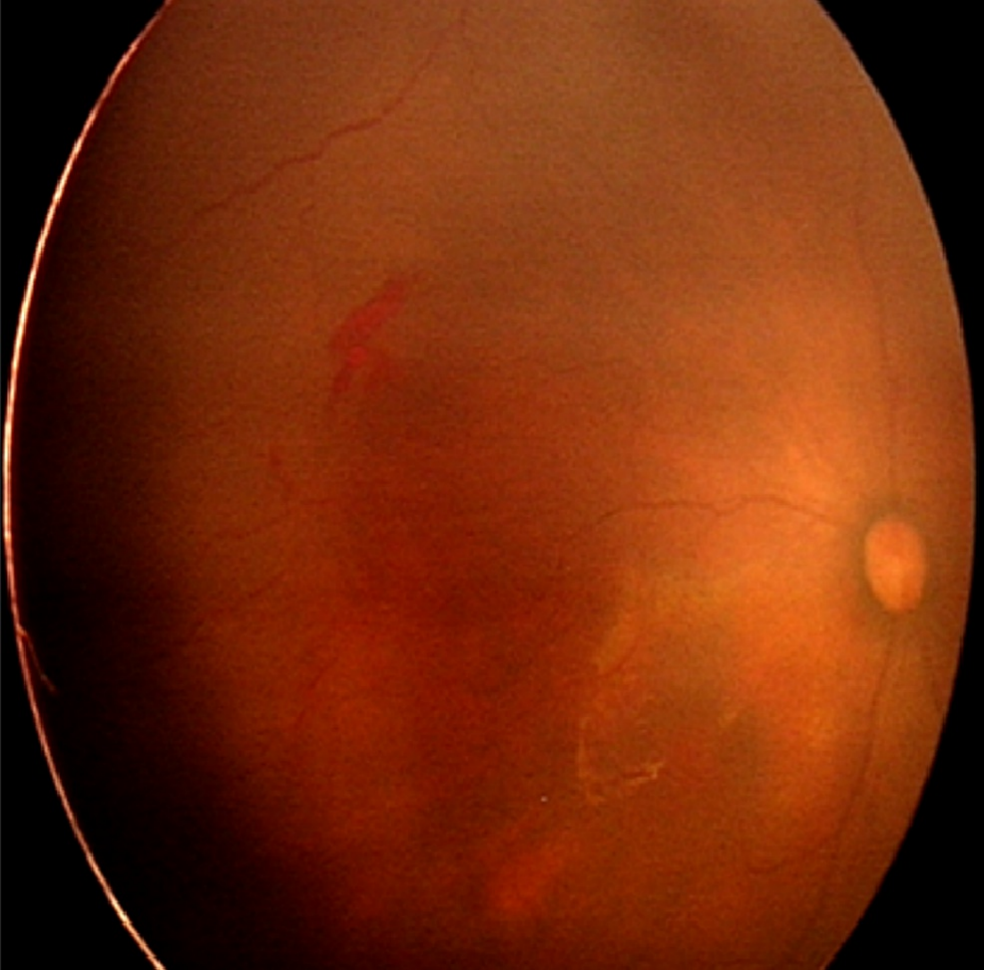
Fundus of the right eye at 3 weeks post-injection showed persistent pre-retinal hemorrhage but smaller in size, vessels were less tortuous and mildly dilated

**Figure 4:**
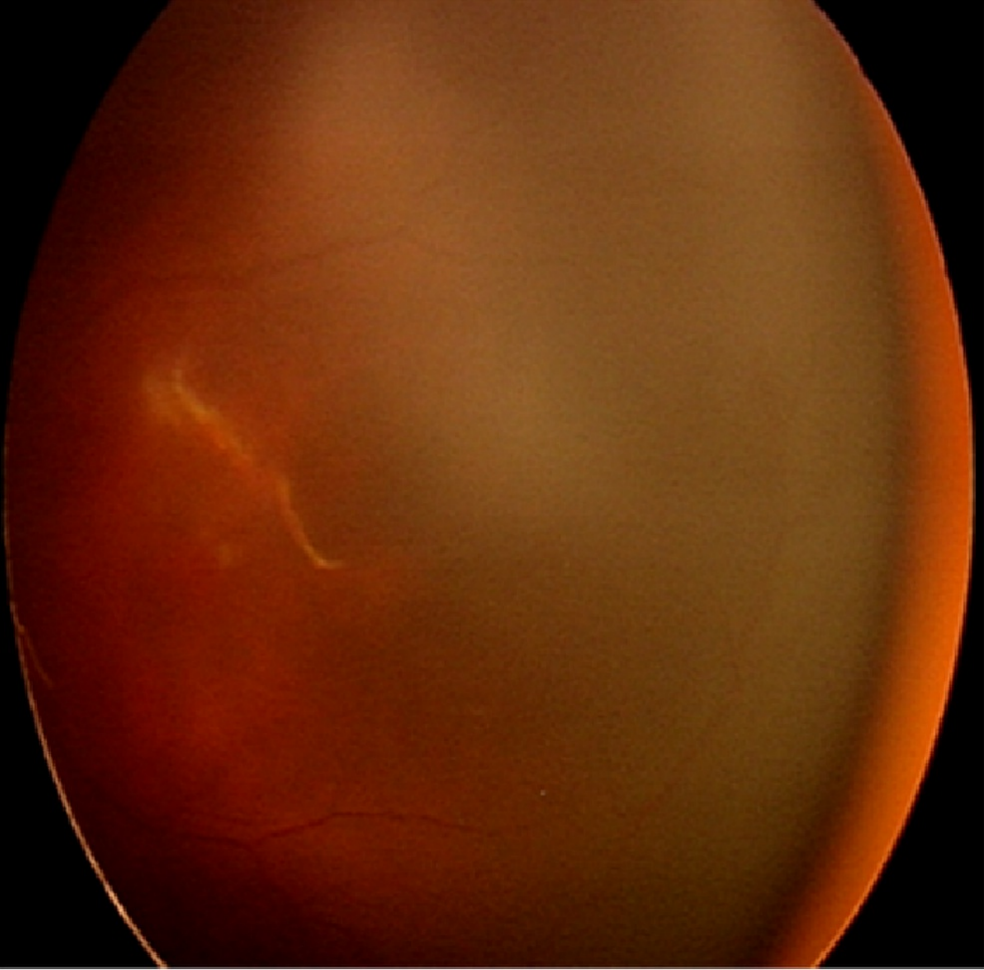
Fundus of the left eye at 3 weeks post-injection showed resolved pre-retinal hemorrhage with less tortuous and less dilated vessels.

A diagnosis of APROP was made and after thorough counseling given to both parents, intravitreal anti-vascular endothelial growth factor (anti-VEGF) was administered. Intravitreal ranibizumab (Alcon, Switzerland) 0.2mg/ 0.02ml was injected at 1.5mm distance from the limbus at inferonasal and inferotemporal of right eye and left eye, respectively, to the sedated infant. Post injection, the baby was started on moxifloxacin 0.5% (Alcon, Singapore) eye drop every six hours. Five days later, fundus examination showed regression of pre-retinal hemorrhage on the left eye with less dilated and less tortuous vessels. Right eye still showed persistent pre-retinal hemorrhage at this time. At 36 weeks CGA, pre-retinal hemorrhage on the right eye had reduced in size and the vessels were less dilated and tortuous, while pre-retinal hemorrhage on the left eye had resolved completely (Figures 3, 4). Nine weeks later, at 44 weeks CGA, the pre-retinal hemorrhage on right eye had completely resolved and vessels had reached zone 3.

## Discussion

A-ROP may lead to poor vision at an early age if not treated promptly. It is crucial to recognize the importance of ROP screening in at-risk premature babies to prevent devastating complications such as blindness. Numerous studies have been done to identify the risk factors associated with A-ROP, formerly known as APROP, which generally occurs in extremely premature and low birth weight infants [9,10]. A study by Flynn et al. proposed the variety of ROP clinical presentation is determined by the disturbance of vasculogenesis phase or angiogenesis phase during embryological development [11]. The vasculogenesis phase is a de novo development of retinal vessel at area of optic disc, while angiogenesis phase is growth of new retinal vessels from existing vessel. Any insult that happens in the vasculogenesis phase would cause disturbance of vasculogenesis and results in a severe zone I aggressive disease [11]. This may happen in a low gestational age baby and early hypoxia. The case that we reported was born premature at 30 weeks gestational age. His birth weight was 1550g which was heavier than the usually proposed high-risk weight for APROP of 1000g. However, a few studies from India have reported APROP occurring in older and heavier infants [12-14]. Shah et al. reported the occurrence of APROP in 99 babies with >28 weeks period of gestation and birth weight (BW) of >1000g [15]. A study by Sanghi et al. reported that APROP in heavier premature infants weighing ≥1500g showed evidence of flat neovascularization mostly occurring in posterior zone 2 with atypical features like large vascular loops [6]. Similar findings were seen in our patient in whom the retinal hemorrhage occurred at posterior zone 2 with dilated vessels and forming loops.

Receipt of supplemental oxygen has been reported to be a significant risk factor associated with APROP [16]. Shah et al. in their study involving very preterm and low birth weight infants reported that supplemental oxygen therapy could be a precipitating factor for APROP in heavier and older infants. However, most infants in the study had multiple co-morbidities which could also have been a contributing factor [15]. Another study involving 15 infants with birth weight of ≥1500g reported that prolonged (≥ three days), unmonitored supplementation of oxygen with presence of other co-morbidities could be a contributing factor toward the development of APROP [6]. Based on the definition in the Early Treatment for ROP (ETROP) study, severe ROP was defined as ROP requiring treatment (photocoagulation or anti-vascular endothelial growth factor) which includes APROP. Another study also showed a relationship between fluctuations of peripheral capillary oxygen saturation (SpO2) in premature infants with development of severe ROP [17]. A fluctuation in oxygen saturation is important to avoid. In our report, the baby received supplemental oxygen since birth. He also had recurrent episodes of apnoea as a result of GERD. High oxygen saturation peaks often occur during bagging of infants following apnea, desaturation, or suctioning.

Intra-amniotic infection or chorioamnionitis have also been reported to be associated with development of APROP. Compared to non-APROP infants, chorioamnionitis was seen more frequently in APROP infants [8]. The American College of Obstetricians and Gynecologists (ACOG) in a workshop has recommended three different categories of intra-amniotic infection. They are 1) confirmed intra-amniotic infection, 2) suspected intra-amniotic infection, and 3) isolated maternal fever [18]. Confirmed intra-amniotic infection is either by evidence of placental pathology or positive test of amniotic fluid. Histologically placenta should show evidence of infection or inflammation, while positive test of amniotic fluid is based on glucose level, gram stain or positive culture [18]. Prior to that, intra-amniotic infection confirmation also includes microbiologic assessment that shows positive for *Mycoplasma hominis* or *Ureaplasma urealyticum* in placental tissue, maternal vaginal swab, or infant gastric juice [19]. Normal in utero growth of retinal vessels may be interfered in the presence of chorioamnionitis especially in premature infants [8]. Besides, *Ureaplasma* colonization in postnatal premature infants was also reported independent risk factor for severe ROP requiring treatment [20]. In our case, the mother had received treatment for chorioamnionitis and the baby had received treatment for *Ureaplasma urealyticum* pneumonia. These could be risk factors for the baby to develop APROP. Ahn et al. reported that intrauterine growth retardation was found to be associated with the occurrence of APROP. Normal in utero retinal vascular growth that is inhibited during perinatal maternal environment may contribute to increasing the risk of APROP [8]. However, the case study in our report was born appropriate for gestational age.

With the improving survival of neonates across the world, there is a likelihood of increasing cases of ROP including APROP, recently addressed as A-ROP. APROP, which was previously observed in more immature infants with extreme low birth weight, is currently seen to develop in bigger and more mature infants, especially in developing countries. Due to its rarity, not many studies have reported the significant risk factors for APROP. It is important to study the risk factors of this disease especially in bigger and more mature infants as more numbers of cases have been reported. In our case, the risk factors involved in the development of APROP were preterm at 30 weeks gestation, receipt of supplemental oxygen, presence of maternal chorioamnionitis and postnatal ureaplasma infection.

## Conclusions

APROP can occur in larger and more mature preterm infants especially if they have received prolonged oxygen supplements and there is history of maternal chorioamnionitis or ureaplasma infection. A high level of vigilance is needed to ensure that APROP/A-ROP is not missed and treatment is constituted promptly.
